# Nitrogen‐Doped Cobalt Pyrite Yolk–Shell Hollow Spheres for Long‐Life Rechargeable Zn–Air Batteries

**DOI:** 10.1002/advs.202001178

**Published:** 2020-10-13

**Authors:** Xue Feng Lu, Song Lin Zhang, Enbo Shangguan, Peng Zhang, Shuyan Gao, Xiong Wen (David) Lou

**Affiliations:** ^1^ School of Chemical and Biomedical Engineering Nanyang Technological University 62 Nanyang Drive Singapore 637459 Singapore; ^2^ School of Materials Science and Engineering Henan Normal University Xinxiang Henan 453007 P. R. China

**Keywords:** cobalt pyrite, nitrogen doping, oxygen electrocatalysis, yolk–shell materials, Zn–air batteries

## Abstract

Limited by the sluggish four‐electron transfer process, designing high‐performance nonprecious electrocatalysts for the oxygen evolution reaction (OER) and oxygen reduction reaction (ORR) is urgently desired for efficient rechargeable Zn–air batteries (ZABs). Herein, the successful synthesis of porous nitrogen‐doped cobalt pyrite yolk–shell nanospheres (N‐CoS_2_ YSSs) is reported. Benefiting from the abundant porosity of the porous yolk–shell structure and unique electronic properties by nitrogen doping, the as‐prepared N‐CoS_2_ YSSs possess more exposed active surface, thus giving rise to superior activity for reversible oxygen electrocatalysis and outstanding cycling stability (more than 165 h at 10 mA cm^−2^) in ZABs, exceeding the commercial Pt/C and RuO_2_ hybrid catalysts. Moreover, the assembled ZABs, delivering a specific capacity of 640 mAh g_Zn_^−1^, can be used for practical devices. This work provides a novel tactic to engineer sulfides as high efficiency and promising bifunctional oxygen electrocatalysts for advanced metal–air batteries.

## Graphical abstract

Nitrogen‐doped cobalt pyrite yolk–shell spheres (N‐CoS_2_ YSSs) are synthesized by a facile two‐step method. The unique morphological architecture and electronic properties endow N‐CoS_2_ YSSs with more exposed active sites and they are thus suitable for rapid charge transfer, resulting in superior activity for reversible oxygen electrocatalysis and outstanding cycling stability in Zn–air batteries.

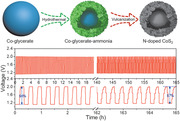

Oxygen electrocatalysis has received widespread attention due to its importance in fuel cells, water splitting, and metal–air batteries.^[^ ^1^, ^2^, ^3^, ^4^ ^]^ Among these sustainable energy storage and conversion technologies, Zn–air batteries (ZABs) have been recognized as a promising global portfolio storage technology due to their unique half‐open systems, significant theoretical energy density (1086 Wh kg^−1^, including oxygen), much flatter operating voltage (1.66 V), environmental benignity, and good safety.^[^ ^3^, ^5^ ^]^ However, the sluggish kinetics of oxygen evolution reaction (OER) for charging and oxygen reduction reaction (ORR) for discharging has been proved to be a complicated bottleneck, significantly leading to high overpotential, low energy efficiency, and limited cycle life.^[^ ^6^, ^7^ ^]^ Noble metal‐based materials are still recognized as the best‐known OER/ORR electrocatalysts (e.g., IrO_2_/RuO_2_ for OER and Pt for ORR), but the material scarcity and high cost severely limit their widespread commercialization. Moreover, their insufficient catalytic bifunctionality and inferior durability fail to fulfill the requirements of promoting reversible OER and ORR simultaneously.^[^ ^6^, ^8^, ^9^ ^]^ Therefore, exploring efficient and robust bifunctional oxygen electrocatalysts is urgently needed but extremely challenging to advance the rechargeability and stability of ZABs.

Currently, many non‐noble metal‐based bifunctional oxygen electrocatalysts, including metal‐free carbon‐based composites, transitional metal oxides, sulfides, carbides, and phosphides have been explored as the air cathodes for ZABs owing to their abundance, cost effectiveness, and comparable electrocatalytic activity.^[^ ^10^, ^11^, ^12^, ^13^, ^14^ ^]^ Among these electrocatalysts, transition metal sulfides, in particular cobalt sulfides, including Co_9_S_8_, CoS, Co_3_S_4_, and CoS_2_, have been widely studied as efficient precatalysts toward OER under alkaline conditions and catalysts for ORR in acidic/alkaline electrolytes.^[^ ^15^, ^16^, ^17^, ^18^ ^]^ Their good electronic conductivity, versatile redox properties, as well as unsaturated transition metal sites are favorable for adsorbing OH^−^ and oxygen‐containing intermediates on the surface. In particular, metallic cobalt pyrite (CoS_2_) shows high intrinsic conductivity for fast charge transfer, which makes it uniquely advantageous as an OER precatalyst.^[^ ^19^, ^20^, ^21^ ^]^ However, the moderate ORR activity, susceptibility to oxidation, and activity decay in alkaline electrolytes severely limit its practical application in long‐life rechargeable ZABs.^[^ ^22^, ^23^ ^]^ Novel and sophisticated strategies are therefore needed to improve its OER and ORR catalytic performance to meet the requirements of high efficiency and long life in practical applications.

It is well known that electrocatalytic performance is largely determined by the number of active sites exposed and the intrinsic activity of each site.^[^ ^24^ ^]^ Therefore, maximizing the exposure of active sites is the first thought to effectively enhance the electrocatalytic activity. In this regard, porous yolk–shell nanostructures are highly desirable due to their attractive properties, such as high specific surface area, abundant interior space, reduced ion‐diffusion path, and large electrolyte–electrode contact area for oxygen adsorption/desorption.^[^ ^25^, ^26^ ^]^ Second, nitrogen doping is considered as an efficient means to enhance the intrinsic activity of each site by optimizing the electronic properties. The lone‐pair electrons around the N atoms are beneficial for better interaction with the reactants to overcome the intrinsic activation barriers.^[^ ^27^, ^28^ ^]^ Moreover, compared with N‐doped carbon, N‐doped transition metal‐based materials are expected to be more efficient for OER/ORR to overcome the intrinsic activation barriers and facilitate reaction kinetics due to their d‐band centers closer to the Fermi levels.^[^ ^15^, ^29^, ^30^ ^]^

With these considerations in mind, we have developed an effective strategy to engineer CoS_2_ with a porous yolk–shell structure and nitrogen doping through a facile hydrothermal and subsequent low‐temperature vulcanization approach. Different from the usage of highly corrosive NH_3_ gas as the common nitrogen source, ammonium hydroxide is used here as both the etchant and nitrogen source, which ensures that the yolk–shell structure of the catalyst remains intact during the post‐annealing process. Owing to the desired morphology and composition, the as‐prepared porous N‐doped CoS_2_ yolk–shell spheres (N‐CoS_2_ YSSs) exhibit superior electrocatalytic performance toward both OER and ORR with a small overpotential gap, rapid kinetics, and long‐term durability, as well as outstanding cycling stability (more than 165 h at 10 mA cm^−2^) in rechargeable ZABs, exceeding the conventional precious‐metal‐based hybrid catalyst (Pt/C||RuO_2_).

The synthetic procedure of N‐CoS_2_ YSSs is illustrated in **Figure** **1**a. First, Co‐glycerate solid spheres (Co‐G SSs) are synthesized by a previously reported method (see the Supporting Information for the experimental details).^[^ ^31^ ^]^ The X‐ray diffraction (XRD) pattern (Figure S1, Supporting Information) with only one broad diffraction peak indicates the amorphous nature of the Co‐G SSs. The field‐emission scanning electron microscopy (FESEM) and transmission electron microscopy (TEM) images verify the solid nature of Co‐G SSs with a smooth surface and an average diameter of about 600 nm (Figure 1b,c). The as‐prepared Co‐G SSs are converted into Co‐glycerate‐ammonia (Co‐G‐A) YSSs via a chemical etching process using ammonium hydroxide as the etching agent. FESEM and TEM images demonstrate the rough surface and void space between the interior solid cores and the outer shells (Figure 1d,e). The diameter of the Co‐G‐A YSSs is slightly reduced to about 560 nm, which is considered as the result of the etching‐induced shrinkage of Co‐G SSs during the formation of the yolk–shell structure. Energy‐dispersive X‐ray (EDX) spectra reveal that the successful introduction of nitrogen in Co‐G‐A YSSs (Figure S2a,b, Supporting Information).

**Figure 1:**
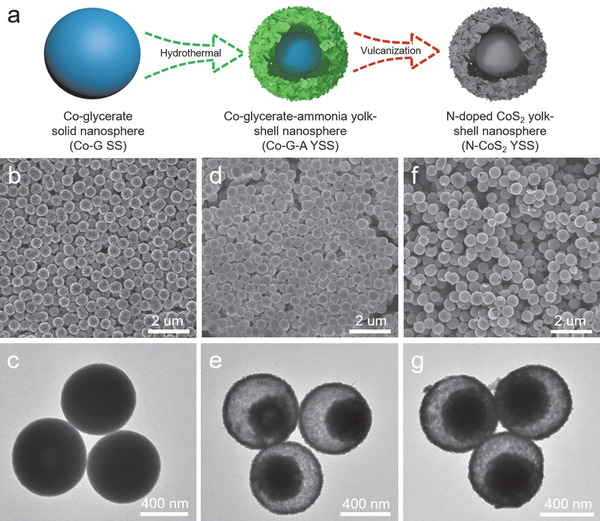
a) Schematic illustration of the preparation for N‐CoS_2_ YSSs. b,d,f) FESEM and c,e,g) TEM images of Co‐G SSs (b,c), Co‐G‐A YSSs (d,e), and N‐CoS_2_ YSSs (f,g).

A subsequent sulfidation process is conducted for the Co‐G‐A YSSs with an appropriate amount of sulfur powder at 300 °C for 2 h with a slow heating rate of 1 °C min^−1^. From a broken nanosphere shown in Figure 1f, a yolk–shell structure can be clearly observed, which is further elucidated by TEM characterization (Figure 1g). EDX analysis validates the presence of Co, S, and N in the N‐CoS_2_ YSSs (Figure S2c, Supporting Information). The doping amount of nitrogen is determined by the elemental analyzer, the atomic ratio of 1/21 for N/S indicates the doping amount of nitrogen is around 0.01 wt%. Moreover, a magnified TEM image of an individual nanosphere unambiguously indicates the void space between the interior solid core and the outer shell (**Figure** **2**a). As can be seen from Figure 2b, the shell of N‐CoS_2_ YSSs is about 30 nm in thickness and constructed by nanoparticles (NPs) with an average size of about 6 nm. This special configuration endows the N‐CoS_2_ YSSs with a substantial number of active sites for oxygen electrocatalysis. The high‐resolution TEM (HRTEM) image (Figure 2c) and the inverse fast Fourier transform (IFFT) images (Figure 2d,e) clearly show two different interplanar spacings of 0.20 and 0.24 nm (Figure S3, Supporting Information), which are readily assigned to the (220) and (210) planes of CoS_2_ (JCPDS card No. 41–1471), respectively. The elemental mapping image shows the homogenous distribution of Co, S, and N elements in N‐CoS_2_ YSSs (Figure 2f). To investigate the spatial distribution of different elements, a line profile and elemental mapping analysis in the scanning TEM (STEM) mode are carried out on an individual N‐CoS_2_ YSS. A high‐angle annular dark field‐STEM image and the corresponding linear scan results imply that the distributions of different elements are quite distinct (Figure S4, Supporting Information). As a comparison, we also prepare porous CoS_2_ SSs by direct vulcanization of Co‐G SSs with sulfur powder (see Supporting Information for details). The morphology and compositional characterizations reveal the successful fabrication of porous CoS_2_ SSs (Figures S5 and S6, Supporting Information).

**Figure 2:**
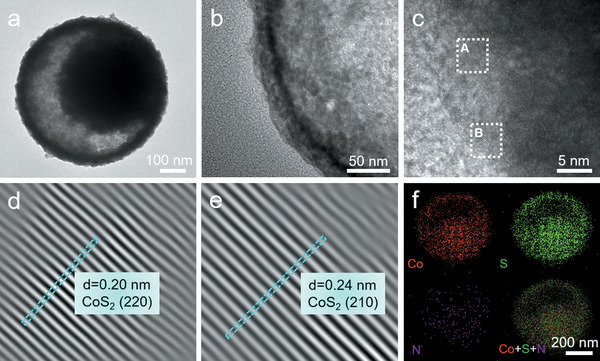
a,b) TEM images of an individual N‐CoS_2_ YSS. c) HRTEM and d,e) the inverse fast Fourier transformation (IFFT) images of the dotted square region A (d) and B (e) in (c). f) The elemental mapping image of an individual N‐CoS_2_ YSS.

XRD analysis (**Figure** **3**a) indicates that the primary crystalline phase is CoS_2_ (JCPDS card No. 41–1471). This suggests that the nitrogen atoms are doped into the lattice sulfur positions of CoS_2_ without destroying the crystal structure. X‐ray photoelectron spectroscopy (XPS) is further employed to analyze the surface chemical states of N‐CoS_2_ YSSs. The survey spectrum (Figure S7, Supporting Information) shows the sample contains C, O, Co, S, and N elements, which is consistent with the EDX result. The peak‐fitting analysis of Co 2p spectrum (Figure 3b) suggests the existence of two chemical states of Co, corresponding to Co—S bonds at 779.2 and 795.4 eV, and Co—O bonds at 781.2 and 797.2 eV. The additional peaks at 784.4 and 801.1 eV could be attributed to the satellite peaks of Co 2p_3/2_ and Co 2p_1/2_, respectively.^[^ ^16^, ^30^ ^]^ The deconvolution of S 2p spectrum (Figure 3c) clearly shows two main peaks that can be subdivided into four peaks, namely, two peaks at 161.5 and 162.6 eV corresponding to the Co—S bonds, and two other peaks at 166.7 and 167.8 eV attributed to the surface oxidized S—O bonds.^[^ ^16^ ^]^ The peak area ratio of 2p_3/2_ to 2p_1/2_ is kept at 2:1. The high‐resolution N 1s spectrum (Figure 3d) can be well deconvoluted into two peaks mainly at 398.1 and 399.7 eV, which are attributed to the Co—N bonds in the lattice matrix and surface oxidized N—O bonds, respectively.^[^ ^30^ ^]^ Nitrogen sorption isotherms (Figure S8, Supporting Information) reveal that the N‐CoS_2_ YSSs have a larger specific surface area (59.7 m^2^ g^−1^) and a broad pore size distribution, which could serve as effective three‐phase interfaces for OER and ORR thus boosting the electrocatalytic performance.^[^ ^32^ ^]^ In short, all these above‐mentioned characterizations and analyses demonstrate the successful design and synthesis of the N‐CoS_2_ YSSs.

**Figure 3:**
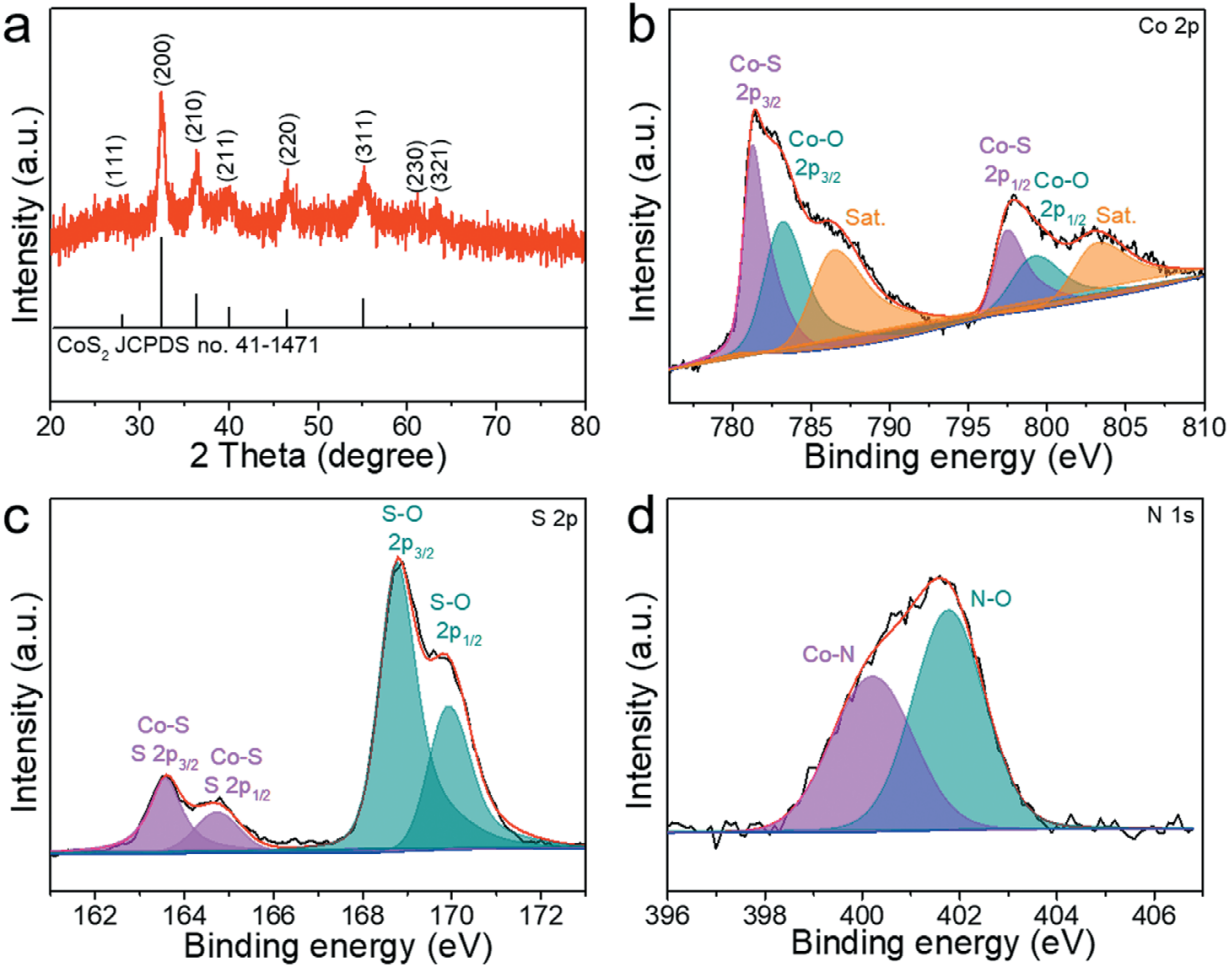
a) XRD pattern and b–d) high‐resolution XPS spectra of Co 2p (b), S 2p (c), and N 1s (d) of N‐CoS_2_ YSSs.

The electrocatalytic performance of CoS_2_ SSs and N‐CoS_2_ YSSs toward OER is first studied by a standard three‐electrode system using linear scan voltammogram (LSV) with *iR* compensation in an alkaline solution (1.0 m KOH), and further benchmarked against the commercial RuO_2_. It can be observed that the N‐CoS_2_ YSSs show better performance toward OER with a lower overpotential than that of the CoS_2_ SSs at the same current densities (**Figure** **4**a). Specifically, the N‐CoS_2_ YSSs only need an overpotential of 278 mV to reach 10 mA cm^−2^ and even smaller overpotentials than that of RuO_2_ at current densities higher than 30 mA cm^−2^. Note that sulfides could undergo the oxidation during OER process, therefore are sometimes called precatalysts.^[^ ^16^, ^33^ ^]^ In addition, the superior OER performance of N‐CoS_2_ YSSs is further supported by its smaller Tafel slope of 56 mV dec^−1^ compared with 64 mV dec^−1^ for CoS_2_ SSs and 83 mV dec^−1^ for RuO_2_ (Figure 4b). The chronopotentiometry test (Figure S9, Supporting Information) reveals that the N‐CoS_2_ YSSs have better stability than RuO_2_ at 10 mA cm^−2^ for 60 h. The present N‐CoS_2_ YSSs are among the best sulfide‐based OER electrocatalysts by comparing their activities (overpotential at 10 mA cm^−2^) and kinetics (Tafel slope) with the reported Co‐based sulfides in the references (Figure 4c and Table S1, Supporting Information), such as CoS_4.6_O_0.6_ (290 mV, 67 mV dec^−1^),^[^ ^34^ ^]^ NiCo_2_S_4_@g‐C_3_N_4_‐CNT (330 mV, 57 mV dec^−1^),^[^ ^35^ ^]^ and Co(S*_x_*Se_1_ *_−x_*)_2_ (280 mV, 66 mV dec^−1^).^[^ ^36^ ^]^

**Figure 4:**
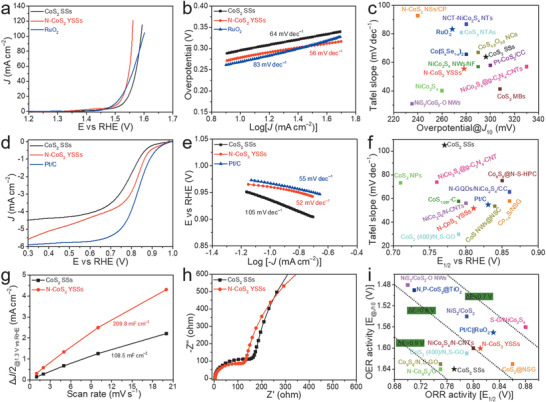
a) LSV curves, b) Tafel slopes of different electrocatalysts toward OER, and c) their activities and kinetics compared with the reported Co‐based sulfides in the references. NSs, nanospheres; CP, carbon paper; NTs, nanotubes; NTAs, nanotube arrays; NCs, nanocubes; NWs, nanowires; NF, nickel foam; CC, carbon cloth; CNTs, carbon nanotubes; MBs, microboxes. d) LSV curves at 1600 rpm, e) Tafel slopes of different electrocatalysts toward ORR, and f) their activities and kinetics compared with the reported Co‐based sulfides in the references. N‐S‐HPC, nitrogen and sulfur co‐doped hollow porous carbon; NSC, nitrogen and sulfur co‐doped carbon; NSG, nitrogen and sulfur co‐doped graphene. g) Half of the capacitive current density difference at 1.3 V versus RHE as a function of scan rates, and h) Nyquist plots for CoS_2_ SSs and N‐CoS_2_ YSSs. i) Comparison of OER and ORR bifunctional activities in this work and the reported catalysts in the references. S‐G, sulfur‐doped graphene. The dotted lines show the Δ*E* at constant values.

An efficient bifunctional electrocatalyst used as the cathode for ZABs should also possess fascinating ORR performance. As observed in Figure 4d, the N‐CoS_2_ YSSs exhibit a more positive onset potential (*η*_0_, 0.95 V) and half‐wave potential (*E*_1/2_, 0.81 V), as well as a larger diffusion‐limiting current density (*J*_d_, 5.6 mA cm^−2^) than those of CoS_2_ SSs, and even comparable to commercial 20 wt% Pt/C (*η*_0_ = 1.0 V, *E*_1/2_ = 0.85 V, *J*_d_ = 5.9 mA cm^−2^), indicating superior ORR performance of N‐CoS_2_ YSSs. The Tafel slope derived from the LSV curve is another key factor to estimate the ORR kinetics. Obviously, the N‐CoS_2_ YSSs possess much faster ORR kinetics (52 mV dec^−1^) compared with CoS_2_ SSs (105 mV dec^−1^), and even faster than Pt/C (55 mV dec^−1^). LSV curves at different rotation speeds (Figure S10, Supporting Information) are used to further investigate the electron transfer kinetics of the ORR process. The Koutecký–Levich (K–L) plots exhibit good linearity and the derived electron transfer number (*n*) is 3.6 for CoS_2_ SSs, 3.7 for N‐CoS_2_ YSSs, and 4.1 for Pt/C, demonstrating a direct four‐electron transfer process during ORR. Besides the catalytic activity, the durability is also evaluated by a chronoamperometry measurement, in which N‐CoS_2_ YSSs show superior durability over Pt/C (Figure S11, Supporting Information). When compared with the sulfide‐based ORR electrocatalysts reported recently (Figure 4f and Table S2, Supporting Information), the as‐obtained N‐CoS_2_ YSSs also show strong competition in terms of activity (*E*_1/2_) and kinetics (Tafel slope), such as CoS_2_ NPs (0.71 V, 73.4 mV dec^−1^),^[^ ^37^ ^]^ CoS_2_(400)/N,S‐GO (0.79 V, 30 mV dec^−1^),^[^ ^23^ ^]^ N‐GQDs/NiCo_2_S_4_/CC (0.86 V, 65.8 mV dec^−1^),^[^ ^17^ ^]^ and Co_9_S_8_@N‐S‐HPC (0.85 mV, 75 mV dec^−1^).^[^ ^38^ ^]^

To understand the promotion of the yolk–shell structure and nitrogen doping on the oxygen electrocatalysis performance, the electrochemically active surface area is evaluated through the electrochemical double‐layer capacitance by measuring the cyclic voltammetry (CV) curves at different scan rates (Figure S12, Supporting Information).^[^ ^39^, ^40^ ^]^ As shown in Figure 4g, the N‐CoS_2_ YSSs show a much higher value (209.8 mF cm^−2^) than that of CoS_2_ SSs (108.5 mF cm^−2^), in line with the BET results, indicating more active sites are exposed in the former. In addition, the electrochemical impedance spectroscopy fitted by an equivalent electrical circuit (Figure S13, Supporting Information) is also used to evaluate the charge transfer kinetics.^[^ ^39^, ^41^ ^]^ The smaller diameter of the semicircle in the high frequency for N‐CoS_2_ YSSs suggests the reduced charge transfer resistance and faster charge transfer kinetics (Figure 4h), which has also been reflected by the fact that N‐CoS_2_ YSSs possess smaller Tafel slopes toward both OER and ORR than CoS_2_ SSs. Based on these analyses, it might be reasonable to propose that the engineering of CoS_2_ with yolk–shell structure and nitrogen doping could offer more exposed active sites and thermodynamically favorable environments, thus boosting the electrocatalytic performance for both OER and ORR.

The overall reversible electrocatalytic ORR/OER performance of all prepared samples is further evaluated by the difference of OER and ORR metrics (Δ*E* = *E_J_*_10_ − *E*_1/2_).^[^ ^42^, ^43^, ^44^ ^]^ Accordingly, the N‐CoS_2_ YSSs display a Δ*E* of 0.79 V, lower than that of CoS_2_ SSs (0.87 V) (Figure S14, Supporting Information). Moreover, this value is also comparable to that of Pt/C||RuO_2_ (0.74 V) and some other reported superior bifunctional electrocatalysts (Figure 4i and Table S3, Supporting Information), such as N,P‐doped CoS_2_@TiO_2_ (0.78 V),^[^ ^30^ ^]^ NiCo_2_S_4_/N‐CNTs (0.80 V),^[^ ^44^ ^]^ and NiS_2_/CoS_2_ (0.75 V),^[^ ^42^ ^]^ demonstrating its potential application in ZABs. A homemade aqueous rechargeable ZAB was assembled according to our previous work (Figure S15, Supporting Information).^[^ ^11^ ^]^ As can be seen in **Figure** **5**a, the battery driven by N‐CoS_2_ YSSs exhibits a stable open‐circuit voltage (1.41 V) for more than 1 h, which is larger than that of CoS_2_ SSs (1.36 V) and comparable to Pt/C||RuO_2_ (1.45 V). A light‐emitting diode (LED) screen could be powered by three batteries in series with N‐CoS_2_ YSSs as the air cathode (inset in Figure 5a). Figure 5b depicts the discharge polarization curves and corresponding power density curves. The battery driven by N‐CoS_2_ YSSs exhibits a maximized power density of 81 mW cm^−2^, higher than that of CoS_2_ SSs (65 mW cm^−2^). Although this value only achieves about 76% of Pt/C||RuO_2_ (107 mW cm^−2^), it surpasses many other reported bifunctional catalysts (Table S4, Supporting Information). The specific capacity of the battery driven by N‐CoS_2_ YSSs reaches up to 744 mAh g_Zn_^−1^ at 5 mA cm^−2^ on the basis of consumed Zn weight (Figure 5c), corresponding to an energy density of 922 Wh kg_Zn_^−1^, which is close to that of Pt/C||RuO_2_ (788 mAh g_Zn_^−1^, 961 Wh kg_Zn_^−1^) but higher than that of CoS_2_ SSs (640 mAh g_Zn_^−1^, 780 Wh kg_Zn_^−1^) and previously reported bifunctional catalysts (Table S5, Supporting Information). Moreover, the cycling rechargeability was further studied at a current density of 10 mA cm^−2^ with a recurrent galvanostatic pulse for 10 min of discharge followed by 10 min of charge (Figure 5d). Remarkably, the battery driven by N‐CoS_2_ YSSs demonstrates superior cycling stability compared to Pt/C||RuO_2_ (Figure 5e,f). For the N‐CoS_2_ YSSs cathode, the voltage fading is negligible with a super stable energy efficiency (discharge end voltage divided by charge end voltage) of 56% after 165 h testing (Figure 5e), reflecting the superior rechargeability. Comparatively, the energy efficiency of the battery driven by Pt/C||RuO_2_ decreases dramatically from 63% to 47% after only 24 h (Figure 5f). The above results verify the promising potential of N‐CoS_2_ YSSs as the air cathode for long‐life rechargeable ZABs.

**Figure 5:**
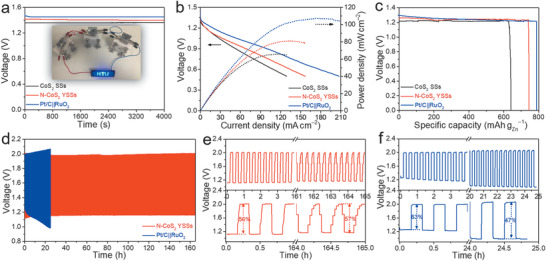
Comparison of performance for ZABs driven by CoS_2_ SSs, N‐CoS_2_ YSSs, and Pt/C||RuO_2_: a) open‐circuit plots (inset is the photograph of a blue LED screen powered by three homemade ZABs in series with N‐CoS_2_ YSSs as the air cathode), b) discharge polarization and power density curves, c) discharge curves at 5 mA cm^−2^, and d–f) long‐term cycling performance at a current density of 10 mA cm^−2^ with: e) N‐CoS_2_ YSSs and f) Pt/C||RuO_2_ as the air cathode.

In summary, we demonstrate that nitrogen‐doped CoS_2_ yolk–shell hollow spheres (N‐CoS_2_ YSSs) as an electrocatalyst exhibit extraordinary activities for both OER and ORR. Starting from the solid cobalt‐glycerate nanospheres, yolk–shelled precursors with nitrogen heteroatoms are first obtained by using ammonium hydroxide as both etchant and nitrogen source. Subsequently, metallic cobalt pyrites doped with nitrogen are formed through low‐temperature vulcanization. This synthesis eliminates the use of highly corrosive NH_3_ gas and high temperature, which not only guarantees the stability of the catalyst structure but also maximizes the exposure of the electrocatalytic active sites. With N‐CoS_2_ YSSs as the air cathode, the Zn–air battery (ZAB) can be stably charged and discharged over 165 h with high energy efficiency, outperforming the more costly Pt/C||RuO_2_‐driven ZABs. This study would inspire the rational design and controllable synthesis of functional chalcogenides and highlight their application prospects in long‐life rechargeable metal–air batteries.

## Conflict of Interest

The authors declare no conflict of interest.

## Supporting information

Supporting Information

## References

[1] N. T. Suen , S. F. Hung , Q. Quan , N. Zhang , Y. J. Xu , H. M. Chen , Chem. Soc. Rev. 2017, 46, 337. 28083578 10.1039/c6cs00328a

[2] M. K. Debe , Nature 2012, 486, 43. 22678278 10.1038/nature11115

[3] Y. Li , H. Dai , Chem. Soc. Rev. 2014, 43, 5257. 24926965 10.1039/c4cs00015c

[4] Z.‐F. Huang , J. Song , S. Dou , X. Li , J. Wang , X. Wang , Matter 2019, 1, 1494.

[5] Y. Li , J. Lu , ACS Energy Lett. 2017, 2, 1370.

[6] H.‐F. Wang , Q. Xu , Matter 2019, 1, 565.

[7] F. Cheng , J. Chen , Chem. Soc. Rev. 2012, 41, 2172. 22254234 10.1039/c1cs15228a

[8] J. Pan , Y. Y. Xu , H. Yang , Z. Dong , H. Liu , B. Y. Xia , Adv. Sci. 2018, 5, 1700691. 10.1002/advs.201700691 PMC5908379 29721418

[9] K. Singh , E. B. Tetteh , H.‐Y. Lee , T.‐H. Kang , J.‐S. Yu , ACS Catal. 2019, 9, 8622.

[10] H. B. Yang , J. Miao , S. F. Hung , J. Chen , H. B. Tao , X. Wang , L. Zhang , R. Chen , J. Gao , H. M. Chen , L. Dai , B. Liu , Sci. Adv. 2016, 2, e1501122. 27152333 10.1126/sciadv.1501122 PMC4846433

[11] X. F. Lu , Y. Chen , S. Wang , S. Gao , X. W. Lou , Adv. Mater. 2019, 31, 1902339. 10.1002/adma.201902339 31348572

[12] H. F. Wang , C. Tang , Q. Zhang , Adv. Funct. Mater. 2018, 28, 1803329.

[13] Z. Cui , Y. Li , G. Fu , X. Li , J. B. Goodenough , Adv. Mater. 2017, 29, 1702385. 10.1002/adma.201702385 28856742

[14] H. Li , Q. Li , P. Wen , T. B. Williams , S. Adhikari , C. Dun , C. Lu , D. Itanze , L. Jiang , D. L. Carroll , Adv. Mater. 2018, 30, 1705796. 10.1002/adma.201705796 29334145

[15] N. Yao , P. Li , Z. Zhou , R. Meng , G. Cheng , W. Luo , Small 2019, 15, 1901993. 10.1002/smll.201901993 31207102

[16] J. Yin , Y. Li , F. Lv , M. Lu , K. Sun , W. Wang , L. Wang , F. Cheng , Y. Li , P. Xi , S. Guo , Adv. Mater. 2017, 29, 1704681. 10.1002/adma.201704681 29239518

[17] W. Liu , B. Ren , W. Zhang , M. Zhang , G. Li , M. Xiao , J. Zhu , A. Yu , L. Ricardez‐Sandoval , Z. Chen , Small 2019, 15, 1903610. 10.1002/smll.201903610 31512394

[18] H. Hu , L. Han , M. Z. Yu , Z. Wang , X. W. Lou , Energy Environ. Sci. 2016, 9, 107.

[19] M. S. Faber , R. Dziedzic , M. A. Lukowski , N. S. Kaiser , Q. Ding , S. Jin , J. Am. Chem. Soc. 2014, 136, 10053. 24901378 10.1021/ja504099w

[20] X. Han , X. Wu , Y. Deng , J. Liu , J. Lu , C. Zhong , W. Hu , Adv. Energy Mater. 2018, 8, 1800935.

[21] Y. Hua , H. Jiang , H. Jiang , H. Zhang , C. Li , Electrochim. Acta 2018, 278, 219.

[22] B. Chen , Z. Jiang , L. Zhou , B. Deng , Z.‐J. Jiang , J. Huang , M. Liu , J. Power Sources 2018, 389, 178.

[23] P. Ganesan , M. Prabu , J. Sanetuntikul , S. Shanmugam , ACS Catal. 2015, 5, 3625.

[24] Z. W. Seh , J. Kibsgaard , C. F. Dickens , I. Chorkendorff , J. K. Norskov , T. F. Jaramillo , Science 2017, 355, eaad4998. 28082532 10.1126/science.aad4998

[25] L. Yu , H. B. Hu , H. B. Wu , X. W. Lou , Adv. Mater. 2017, 29, 1604563.

[26] P. Zhang , B. Y. Guan , L. Yu , X. W. Lou , Angew. Chem., Int. Ed. 2017, 56, 7141. 10.1002/anie.201702649 28488748

[27] D. Guo , R. Shibuya , C. Akiba , S. Saji , T. Kondo , J. Nakamura , Science 2016, 351, 361. 26798009 10.1126/science.aad0832

[28] Y. Zhao , R. Nakamura , K. Kamiya , S. Nakanishi , K. Hashimoto , Nat. Commun. 2013, 4, 2390. 23979080 10.1038/ncomms3390

[29] J. Hao , W. Yang , Z. Peng , C. Zhang , Z. Huang , W. Shi , ACS Catal. 2017, 7, 4214.

[30] L. Guo , J. Deng , G. Wang , Y. Hao , K. Bi , X. Wang , Y. Yang , Adv. Funct. Mater. 2018, 28, 1804540.

[31] L. Shen , L. Yu , X. Y. Yu , X. Zhang , X. W. Lou , Angew. Chem., Int. Ed. 2015, 54, 1868. 10.1002/anie.201409776 25522266

[32] Z. Lu , W. Xu , J. Ma , Y. Li , X. Sun , L. Jiang , Adv. Mater. 2016, 28, 7155. 27296111 10.1002/adma.201504652

[33] Y. Guo , T. Park , J. W. Yi , J. Henzie , J. Kim , Z. Wang , B. Jiang , Y. Bando , Y. Sugahara , J. Tang , Y. Yamauchi , Adv. Mater. 2019, 31, 1807134. 10.1002/adma.201807134 30793387

[34] P. Cai , J. Huang , J. Chen , Z. Wen , Angew. Chem., Int. Ed. 2017, 56, 4858. 10.1002/anie.201701280 28345283

[35] X. Han , W. Zhang , X. Ma , C. Zhong , N. Zhao , W. Hu , Y. Deng , Adv. Mater. 2019, 31, 1808281. 10.1002/adma.201808281 30873660

[36] L. Fang , W. Li , Y. Guan , Y. Feng , H. Zhang , S. Wang , Y. Wang , Adv. Funct. Mater. 2017, 27, 1701008.

[37] C. Zhao , D. Li , Y. Feng , J. Mater. Chem. A 2013, 1, 5741.

[38] S. Zhang , D. Zhai , T. Sun , A. Han , Y. Zhai , W.‐C. Cheong , Y. Liu , C. Su , D. Wang , Y. Li , Appl. Catal., B 2019, 254, 186.

[39] Z. P. Wu , X. F. Lu , S. Q. Zang , X. W. Lou , Adv. Funct. Mater. 2020, 30, 1910274.

[40] X. F. Lu , L. Yu , X. W. Lou , Sci. Adv. 2019, 5, eaav6009. 30793034 10.1126/sciadv.aav6009 PMC6377276

[41] X. F. Lu , L. F. Gu , J. W. Wang , J. X. Wu , P. Q. Liao , G. R. Li , Adv. Mater. 2017, 29, 1604437. 10.1002/adma.201604437 27865016

[42] Y. Cao , X. Zheng , H. Zhang , J. Zhang , X. Han , C. Zhong , W. Hu , Y. Deng , J. Power Sources 2019, 437, 226893.

[43] W. Liu , J. Zhang , Z. Bai , G. Jiang , M. Li , K. Feng , L. Yang , Y. Ding , T. Yu , Z. Chen , A. Yu , Adv. Funct. Mater. 2018, 28, 1706675.

[44] X. Han , X. Wu , C. Zhong , Y. Deng , N. Zhao , W. Hu , Nano Energy 2017, 31, 541.

