# Supplementary material for: Nitrogen‐Doped Cobalt Pyrite Yolk–Shell Hollow Spheres for Long‐Life Rechargeable Zn–Air Batteries
Source: Adv Sci (Weinh). 2020 Oct 13;7(22):2001178. doi: 10.1002/advs.202001178 (PMC7675189; doi:10.1002/advs.202001178)
Supplement: Supplementary file 1 — Supporting Information [file ADVS-7-2001178-s001.pdf]

Supporting Information *for*

Nitrogen-Doped Cobalt Pyrite Yolk–Shell Hollow Spheres for Long-Life Rechargeable Zn–Air Batteries

*Xue Feng Lu, Songlin Zhang, Enbo Shangguan, Peng Zhang, Shuyan Gao,**

*and Xiong Wen (David) Lou**

[*] Dr. E. Shangguan, Prof. S. Y. Gao

Schoof of Materials Science adn Engineering, Henan Normal University, Xinxiang, Henan 453007, P.R. China. Email: shuyangao@htu.cn

Dr. X. F. Lu, S. L. Zhang, Dr. P. Zhang, Prof. X. W. Lou

School of Chemical and Biomedical Engineering, Nanyang Technological University, 62 Nanyang Drive, Singapore, 637459, Singapore

Email: xwlou@ntu.edu.sg; Webpage: http://www.ntu.edu.sg/home/xwlou/

Experimental details

Chemicals. Cobalt nitrate hexahydrate, glycerol, isopropanol, ethanol, ammonia solution (15 wt%), sulfur powder, potassium hydroxide, and zinc acetate dihydrate are purchased from Sigma-Aldrich. All the chemicals were directly used without further purification. Deionized water is used in all experiments.

Synthesis of Co-glycerate solid nanospheres (Co-G SSs). According to our previously reported literature with slight modification.[1,2] In a typical synthesis, 0.37 mmol of $Co(NO_3)_2{\cdot}6H_2O$ was dissolved into a mixture solution of glycerol (8 mL) and isopropanol (40 mL), after stirring for 40 min at 800 rpm, the as-prepared solution was then transferred to a Teflon-lined stainless steel autoclave and kept at 180°C for 6 h. After cooling to room temperature naturally, the brown precipitates were separated using centrifugation, washed five times with ethanol and dried at 80°C.

Synthesis of Co-glycerate-ammonia yolk-shell nanospheres (Co-G-A YSSs). In a typical synthesis, 50 mg of Co-G SSs were dissolved into 20 mL of ethanol followed with 3 mL of ammonia solution (15 wt%), after stirring for 30 min at 800 rpm, the as-prepared solution was then transferred to a Teflon-lined stainless steel autoclave and kept at 160°C for 1 h. After cooling to room temperature naturally, the black precipitates were separated using centrifugation, washed five times with ethanol and dried at 60°C.

Synthesis of porous N-doped CoS_2 (N-CoS_2) YSSs. In a typical synthetic procedure, 20 mg of Co-G-A YSSs and 50 mg of sulfur powders were put at two separate positions in a porcelain boat with sulfur powders at the upstream side of the tube furnace. Then, the samples were annealed at 300°C for 2 h with a heating rate of 1 °C min^{-1} under nitrogen atmosphere. The porous N-CoS_2 YSSs were obtained after cooling to ambient temperature under nitrogen atmosphere.

Synthesis of porous CoS_2 SSs. In a typical synthetic procedure, 20 mg of Co-G SSs and 50 mg of sulfur powders were put at two separate positions in a porcelain boat with sulfur powders at the upstream side of the tube furnace. Then, the samples were annealed at 300°C for 2 h with a heating rate of 1 °C min^{-1} under nitrogen atmosphere. The porous CoS_2 SSs were obtained after cooling to ambient temperature under nitrogen atmosphere.

Material characterizations. The morphology and structure of the products were characterized using field-emission scanning electron microscope (FESEM; JEOL-6700F) equipped with energy-dispersive X-ray spectroscopy (EDX), and transmission electron microscope (TEM; JEOL, JEM-1400/JEM-2100F) equipped with EDX. The high-resolution transmission electron microscope (HRTEM) images were collected on another transmission electron microscope (TEM; JEOL 2010 UHR). X-ray diffractometer (XRD) patterns were collected on a Bruker D2 Phaser with Ni-filtered Cu Ka radiation (λ = 1.5406 Å) at a voltage of 30 kV and a current of 10 mA. The doping amount of nitrogen is measured by the elemental analyser (Vario EL III CHNS Elemental Analyser). The chemical states of the elements on the surface of the samples was evaluated by the X-ray photoelectron spectrometer (XPS; ESCALAB 250). All XPS data were corrected using the C 1s line at 284.6 eV, and curve fitting and background subtraction were accomplished. Nitrogen adsorption measurements were performed in an ASAP 2010 Micromeritics apparatus. The samples were

evacuated at 453 K for 24 h before analysis. The specific surface area was calculated from the N_2 desorption curve by the Barrett-Joyner-Halenda (BJH) method.

Electrochemical measurements. All the electrochemical tests were performed on a CHI 760E electrochemical workstation (CH Instruments, China) with a standard three-electrode system at room temperature. A Pt wire and a Hg/HgO electrode were used as the counter electrode and reference electrode, respectively. The working electrode was prepared as follows: 10 mg of catalysts were firstly dispersed in a mixed solution of water (250 μL), ethanol (750 μL) and 5 wt% Dupont Nafion 117 solution (20 μL), followed by 30 min sonication to form a homogeneous suspension. 10 μL of suspension was pipetted onto a polished rotating disk electrode (RDE, diameter: 5 mm) and subjected to overnight solvent evaporation in air. In the electrochemical testing, the measured potentials were calibrated with respect to reversible hydrogen electrode (RHE) according to the Nernst equation: E (V vs RHE) = E (V vs Hg/HgO) + 0.098 + 0.0592 × pH, and the current density was normalized to the effective geometrical surface area (0.196 cm^2). The linear scan voltammetry (LSV) curves were performed at a scan rate of 5 mV s^{-1}. The electrochemical active surface area (ECSA) of the catalysts was evaluated by measuring the double-layer capacitance (C_{dl}) under a potential window without redox reactions. The electrochemical impedance spectroscopy (EIS) was carried out at the open circuit potential with a frequency range from 10^{-2} Hz to 10^{5} Hz. The OER was tested in 1.0 M KOH solution and ORR was measured in 0.1 M KOH solution saturated with oxygen. The electron transfer number (n) and the kinetic current densities (J_K) were determined from the Koutecky-Levich (K-L) equation expressed as follows:

$$1/J = 1/J_K + 1/(B\omega^{1/2})$$

where J, J_K, and ω are the measured current density, kinetic limiting current density and rotating rate. B is determined from the Levich slope as given below:

$$B = 0.2\ n\ F\ C_0\ D_0^{2/3}\ v^{-1/6}$$

in which F is the Faraday constant (F = 96485 C mol^{-1}). C_0 is the concentration of O_2 in the solution ($C_0 = 1.2 \times 10^{-6}$ mol cm^{-3}). D_0 is the diffusion coefficient of O_2 in 0.1 M KOH ($D_0 = 1.9 \times 10^{-5}$ cm^2 s^{-1}), and v is the kinematic viscosity of the electrolyte (v = 0.01 cm^2 s^{-1}). The constant of 0.2 is adopted when rotating speed is expressed in rpm.

Aqueous Zn-air battery assembly. A home-made aqueous zinc-air battery was employed to test the traditional stack-type battery performance. A polished zinc foil (thickness: 0.3 mm, area: 1×5 cm^2) was used as the anode. A hydrophilic carbon fiber paper substrate with a gas diffusion layer on the air-facing side and a catalyst layer on the water-facing side was used as the air cathode. The catalyst ink was sprayed onto the carbon fiber paper to obtain a mass loading of 10 mg cm^{-2}. The air cathode had an effective contact area of 1 cm^2 to the electrolyte and air. A mixed solution of 6.0 M KOH + 0.2 M $Zn(CH_3COO)_2{\cdot}2H_2O$ was used to ensure reversible zinc electrochemical reactions at the Zn anode. Measurements were carried out at room temperature with an electrochemical workstation (CHI 760E, CH Instrument). The polarization curve measurements were performed by LSV at a scan tare of 5 mV s^{-1}. Cycling test was performed using recurrent galvanostatic pulses for 10 min of discharge followed by 10 min of charge at 10 mA cm^{-2}. Both the current density and power density were normalized to the effective surface area of the air electrode. The specific capacity was calculated from the galvanostatic discharge curve, normalized to the mass of consumed Zn anode.

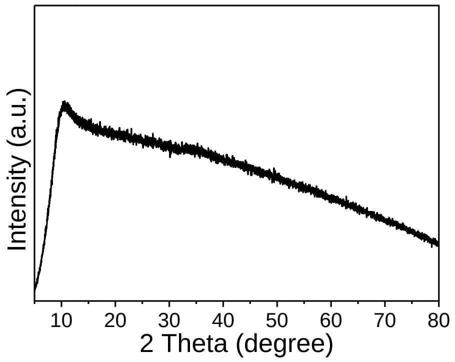

Figure S1. XRD pattern of Co-G SSs.

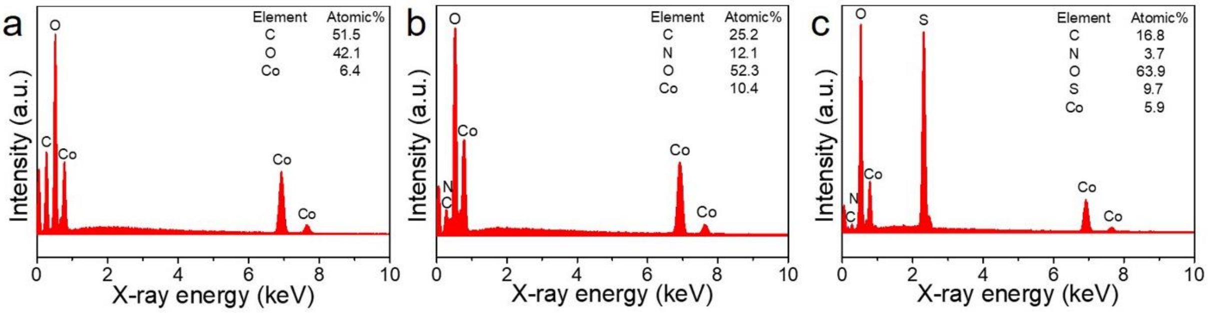

Figure S2. EDX spectra of (a) Co-G SSs, (b) Co-G-A YSSs, and (c) N-CoS_2 YSSs.

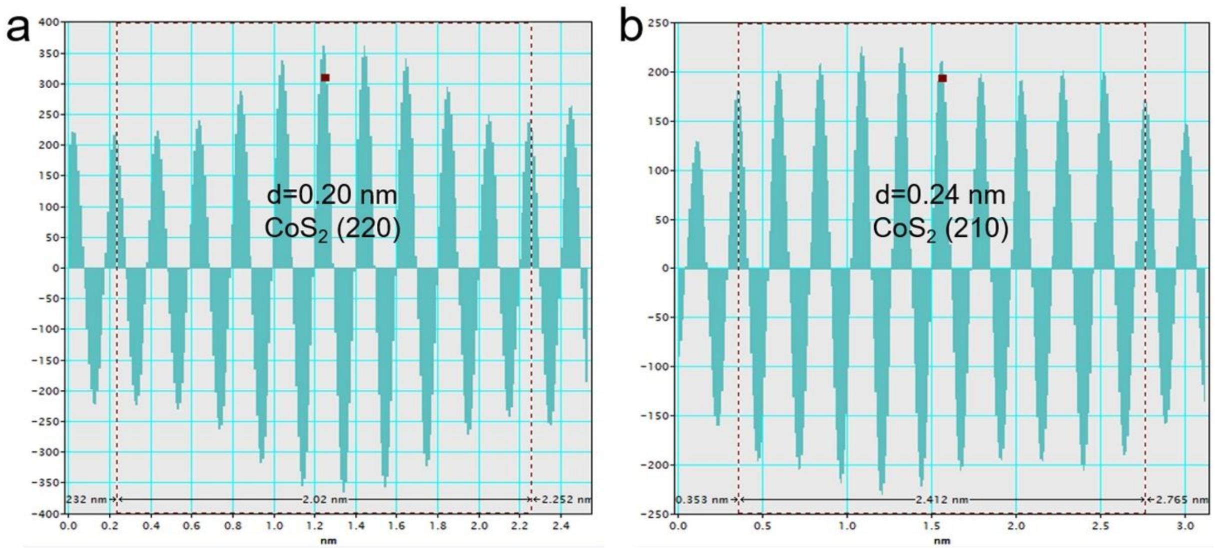

Figure S3. The corresponding line scan results of the dotted square region A (a) and B (b) in Figure 2c.

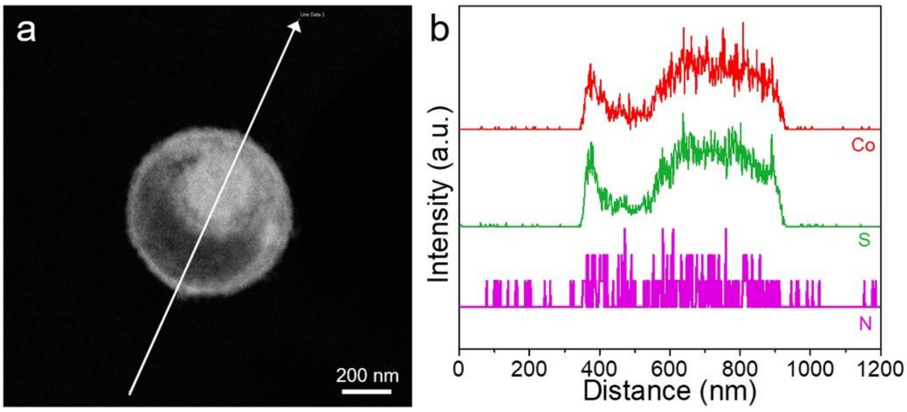

Figure S4. (a) High-angle annular dark-field scanning transmission electron microscopy (HAADF-STEM) of an individual N-CoS_2 YSS and (b) linear elemental distributions of Co, S, and N, corresponding to the white arrow in (a).

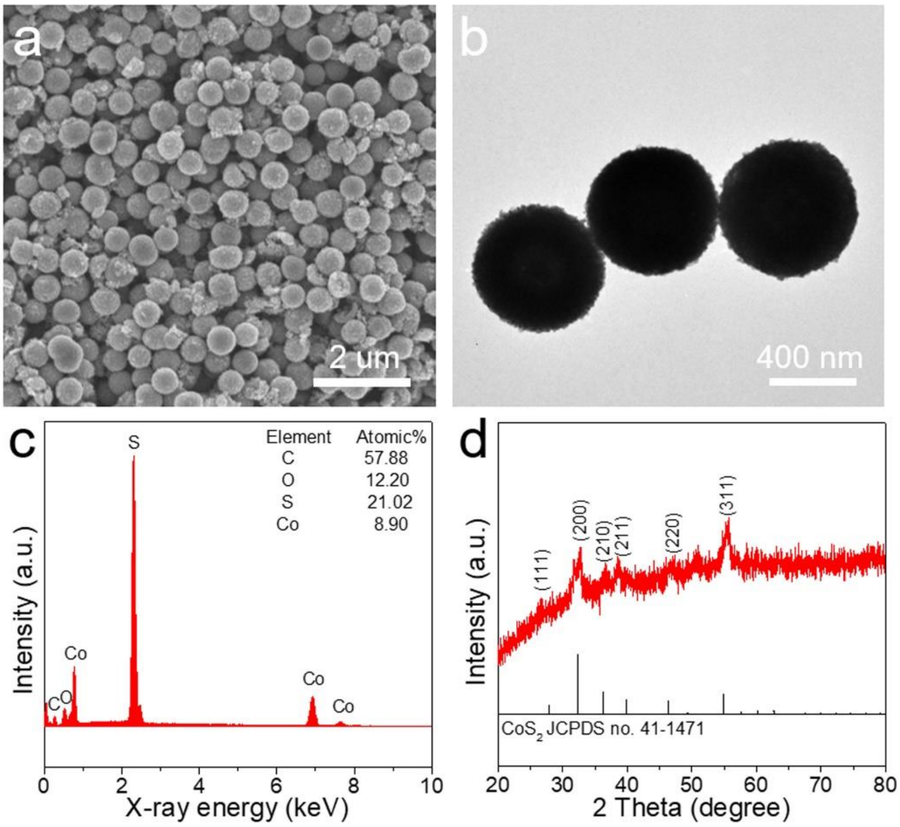

Figure S5. (a) FESEM image, (b) TEM image, (c) EDX spectrum, and (d) XRD pattern of porous CoS_2 SSs.

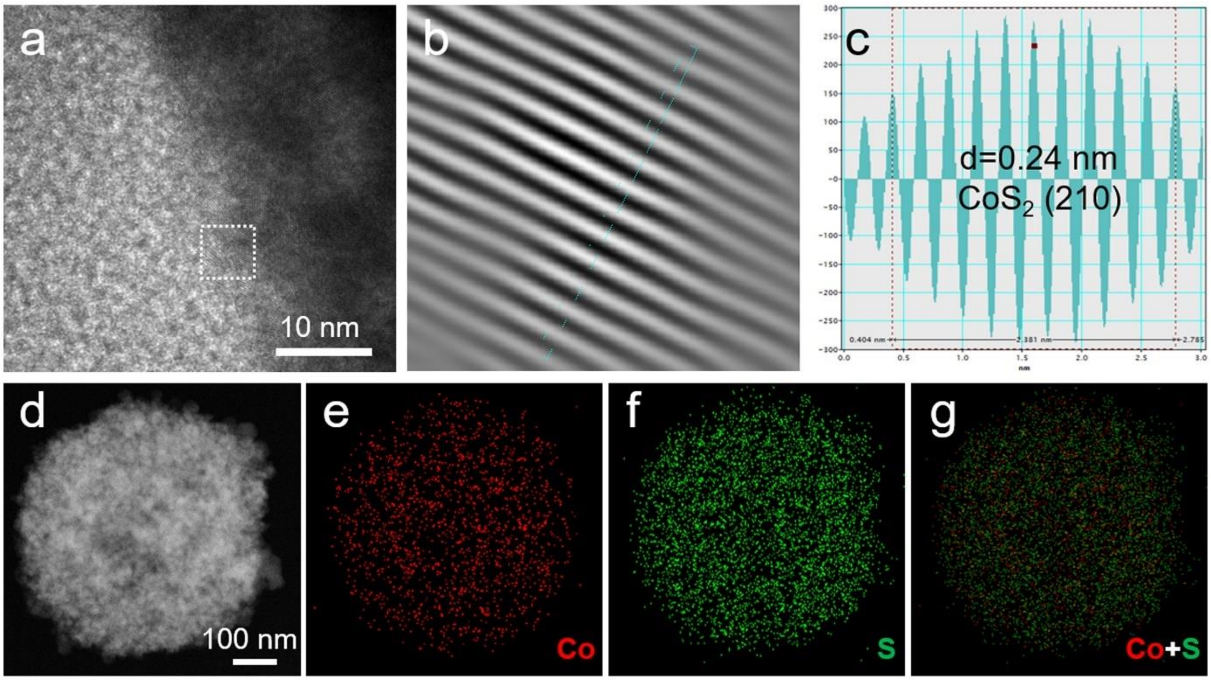

Figure S6. (a) HRTEM image of CoS_2 SSs; (b) the inverse fast Fourier transformation (IFFT) image, and (c) corresponding line scan of the dotted square region in figure a. (d) HAADF-STEM image, (e-g) elemental mappings of an individual CoS_2 SS.

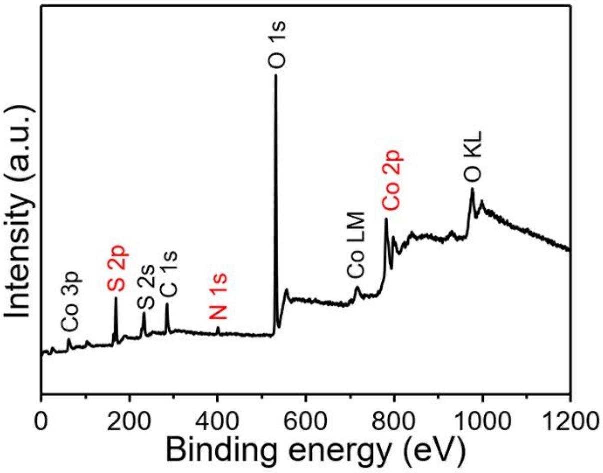

Figure S7. XPS survey spectrum of N-CoS_2 YSSs.

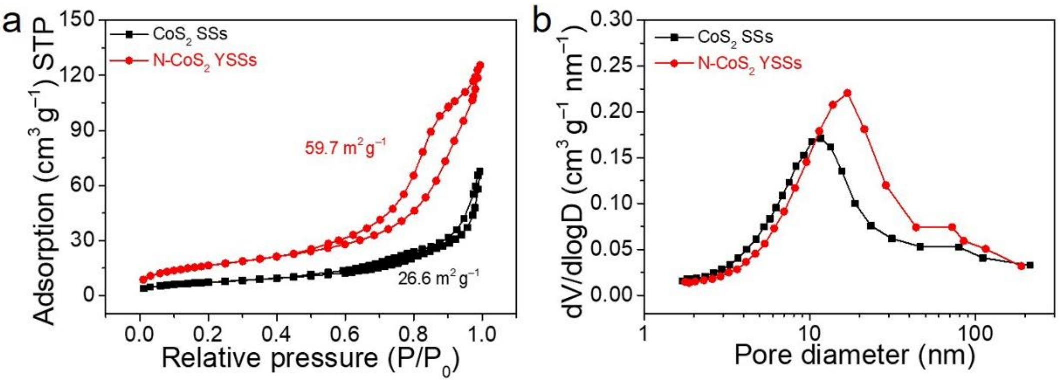

Figure S8. (a) N_2 sorption isotherms and (b) the corresponding pore size distribution of CoS_2 SSs and N-CoS_2 YSSs.

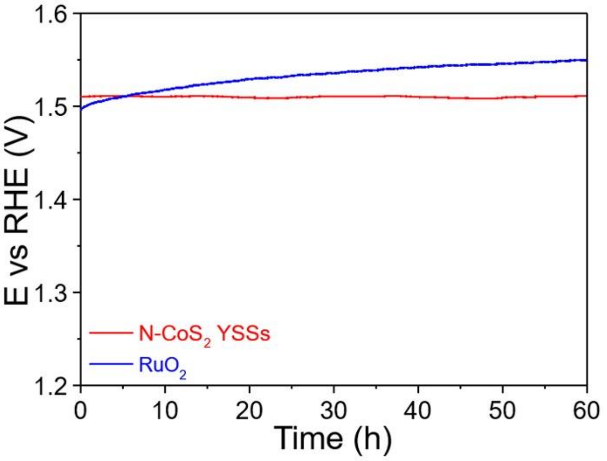

Figure S9. Chronopotentiometry curves at a constant current density of 10 mA cm^{-2} for N-CoS_2 YSSs and RuO_2.

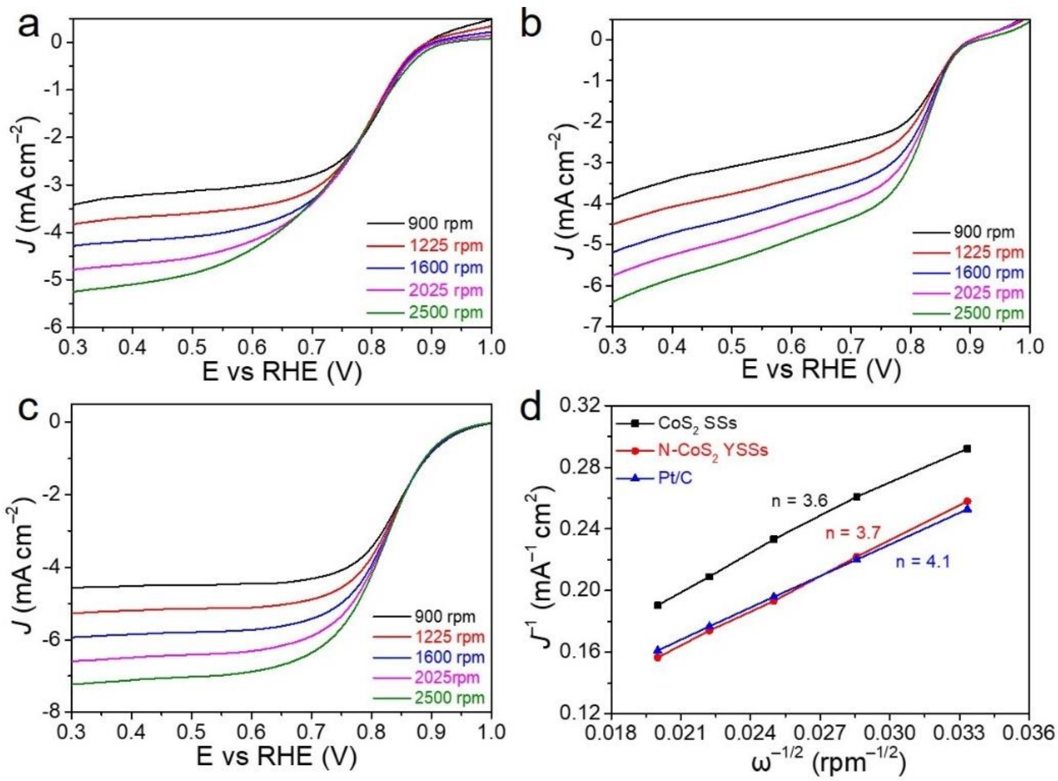

Figure S10. LSV curves on RDE in O_2-saturated 0.1 M KOH solution at different rotation speeds from 900 to 2500 rpm at a scan rate of 5 mV s^{-1} for (a) CoS_2 SSs, (b) N-CoS_2 YSSs, and (c) Pt/C (20 wt%). (d) The K-L plots at 0.3 V vs RHE, showing the electron transfer number of different samples.

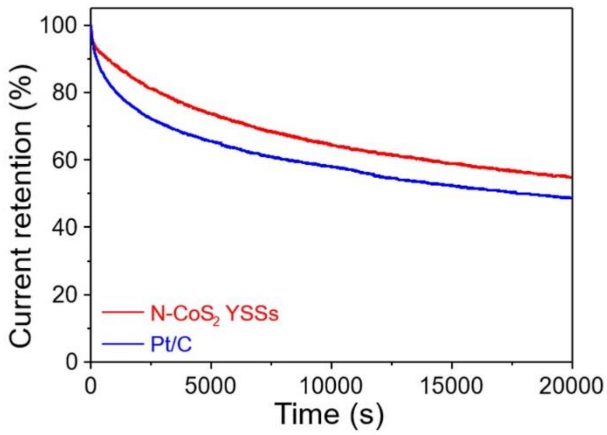

Figure S11. Chronoamperometry curves of N-CoS_2 YSSs and Pt/C at 0.77 V in O_2-saturated 0.1 M KOH solution.

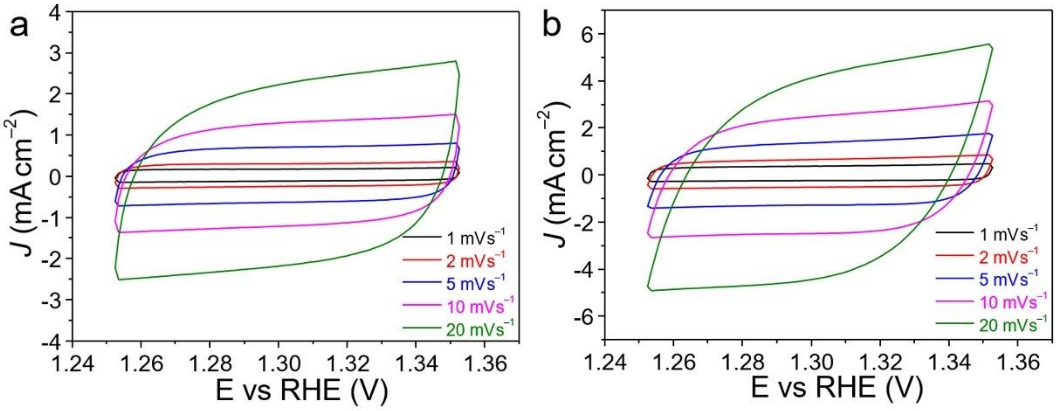

Figure S12. CV curves at different scan rates for (a) CoS_2 SSs and (b) N-CoS_2 YSSs.

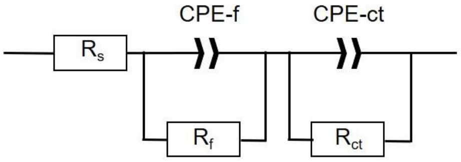

Figure S13. The equivalent circuit consisting of a solution resistance (R_s) in series with two parallel branches: one reflecting the charge-transfer process (CPE-ct) and the other related to the surface porosity (CPE-f).

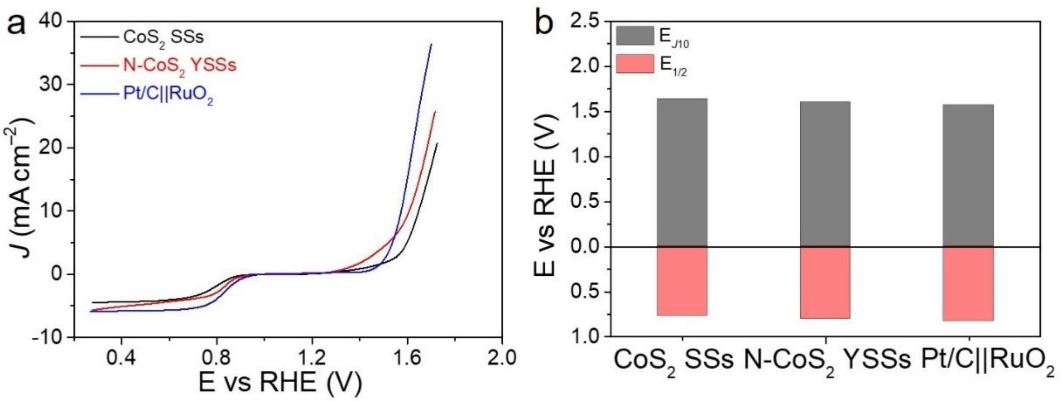

Figure S14. (a) The overall polarization curves of catalysts within the ORR and OER potential window, and (b) the histogram about the values of E_{J10} for OER and $E_{1/2}$ for ORR.

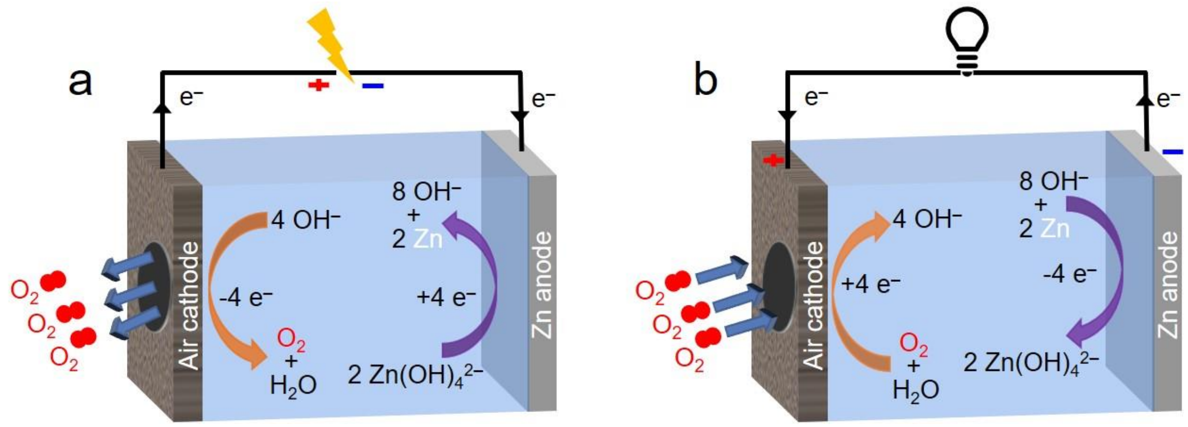

Figure S15. Schematic configuration of the Zn-air batteries during (a) charging and (b) discharging processes.

Table S1. Comparison of the OER performance of N-CoS_2 YSSs with some representative Co-based sulfides reported in 1.0 M KOH solution.

Catalysts	$\eta@J$ (mV@mA cm^{-2})	Tafel slope (mV dec^{-1})	Reference
N-CoS_2 YSSs	278@10<br>297@20	56	This work
$NiCo_2S_4$ NWs/NF	260@10	40	[3]
CoS_2 NTAs	276@10	81	[4]
NiS_2/CoS_2-O NWs	235@10	31	[5]
N-CoS_2 NS/CP	240@10	93	[6]
$CoS_{4.6}O_{0.6}$ NCs	290@10	67	[7]
$Co(S_xSe_{1-x})_2$	280@10	66	[8]
Pt-CoS_2/CC	300@10	58	[9]
CoS_2 MBs	308@10	41	[10]
CoS_2 NSs	290@10	57	[11]
N-CoS_2/NF	200@20	55	[12]
$NiCo_2S_4$@g-C_3N_4-CNT	330@10	57	[13]
NiS_2/CoS_2/C	310@20	78	[14]
100-NCT-$NiCo_2S_4$ NTs	280@10	87	[15]
Co−WS_2	303@10	79	[16]
CoS/NSC	340@10	97	[17]
NiCoDH/NiCoS	303@20	77.6	[18]
$Co_{0.37}S_{0.38}P_{0.02}$	257@10	44	[19]
Co_3S_4	270@10	45.4	[20]

Table S2. Comparison of the electrocatalytic activities of N-CoS_2 YSSs with some representative sulfides-based ORR electrocatalysts reported in 0.1 M KOH solution.

Catalysts	E_o (V)	$E_{1/2}$ (V)	Tafel slope (mV dec^{-1})	Reference
N-CoS_2 YSSs	0.95	0.81	52	This work
$Co_{1-x}S$/RGO	0.87	0.76[a]	N.A.	[21]
CoS_2 NPs	0.94	0.71	73.4	[22]
CoS_2(400)/N,S-GO	0.97	0.79	30	[23]
$FeNiS_2$ sheets	0.78	0.64	107	[24]
Co-C@Co_9S_8 DSNCs	0.96	0.83	N.A.	[25]
$NiCo_2S_4$/N-CNTs	0.93	0.80	56	[26]
NiS_2/CoS_2-O NWs	0.85	0.70	N.A.	[5]
$Co_{1-x}S$/NSG	0.98	0.86	58	[27]
S-GNS/$NiCo_2S_4$	0.93[a]	0.88	N.A.	[28]
N,P-doped CoS_2@TiO_2	0.91	0.71	N.A.	[29]
CoS_2@NSG	0.96	0.86	N.A.	[30]
CoS NWs@NSC	0.93	0.84	54	[31]
N-GQDs/$NiCo_2S_4$/CC	0.97	0.86	66	[32]
$NiCo_2S_4$@g-C_3N_4-CNT	0.87	0.76	74	[13]
NiS_2/CoS_2	0.90	0.79	N.A.	[33]
Co_9S_8@N-S-HPC	0.99	0.85	75	[34]
$CoS_{1.097}$-C	0.90	0.79	58	[35]
Co−WS_2	0.95[a]	0.84	114	[16]
$NiCoMnS_4$/N-RGO	0.94	0.81	N.A.	[36]
$NiCo_2S_4$/$RGO_{0.02}$	0.90[a]	0.78	N.A.	[37]
CoS/NSC	0.94	0.84	N.A.	[17]
Co-Ni-S@NSPC	0.95	0.82	N.A.	[38]
Co_9S_8/C	0.89	0.78	N.A.	[39]

[a] This value is not mentioned in the literature but derived from the LSV curve.

Table S3. Comparison of the electrocatalytic activities of N-CoS_2 YSSs and some representative sulfide-based bifunctional electrocatalysts reported in 0.1 M KOH solution.

Catalysts	E_{J10} (V)	$E_{1/2}$ (V)	ΔE (E_{J10} - $E_{1/2}$) (V)	Reference
N-CoS_2 CSSs	1.60	0.81	0.79	This work
CoS_2(400)/N,S-GO	1.61	0.79	0.82	[23]
Co_9S_8/N,S-GO	1.63	0.75	0.88	[23]
N-doped Co_9S_8/G	1.64	0.75[a]	0.89	[40]
NiS_2/CoS_2-O NWs	1.48	0.70	0.78	[5]
$NiCo_2S_4$/N-CNTs	1.60	0.80	0.80	[26]
$CuCo_2S_4$ nanosheets	1.52	0.69	0.83	[41]
CoS_2@NSG	1.63	0.86	0.77	[30]
N,P-doped CoS_2@TiO_2	1.49	0.71	0.78	[29]
S-GNS/$NiCo_2S_4$	1.56	0.88	0.68	[28]
NiS_2/CoS_2	1.54	0.79	0.75	[33]

[a] This value is not mentioned in the literature but derived from the LSV curve.

Table S4. Comparison of the power density of Zn-air batteries driven by CoS_2 SSs, N-CoS_2 YSSs and Pt/C||RuO_2 with recently reported Zn-air batteries.

Catalysts	Electrolyte	Power density (mW cm^{-2})	Reference
CoS_2 SSs		65	
N-CoS_2 YSSs	6.0 M KOH + 0.20 M $Zn(Ac)_2$	81	This work
Pt/C||RuO_2		107	
CoS_2@N-S-G	6 M KOH	151	[30]
N-GQDs/$NiCo_2S_4$/CC	6.0 M KOH + 0.20 M $Zn(Ac)_2$	75.2	[32]
$NiCo_2S_4$/N-CNTs	6.0 M KOH + 0.20 M $ZnCl_2$	147	[26]
$NiCo_2S_4$@g-C_3N_4-CNT	6.0 M KOH + 0.20 M $ZnCl_2$	142	[13]
Co-N,B-CSs	6.0 M KOH + 0.20 M $Zn(Ac)_2$	100.4	[42]
CoP NCs	3.0 M KOH + 0.20 M $Zn(Ac)_2$	66.7	[43]
200-CNTs-Co/NC	6.0 M KOH + 0.20 M $ZnCl_2$	83.1	[44]
FeNi-NC	6 M KOH + 0.2 M ZnO	80.8	[45]
Co_3O_4-doped Co/CoFe	6 M KOH	97	[46]
Co/Co_3O_4@PGS	6.0 M KOH + 0.20 M $Zn(Ac)_2$	118	[47]
Co-POC	6.0 M KOH + 0.20 M $ZnCl_2$	78	[48]
Pt/C + Ir/C	6.0 M KOH + 0.20 M $ZnCl_2$	73.4	[49]

Table S5. Comparison of the specific capacity and energy density of Zn-air batteries driven by CoS_2 SSs, N-CoS_2 YSSs and Pt/C||RuO_2 with recently reported Zn-air batteries.

Catalysts	Specific capacity (mAh g_{Zn}^{-1})	Energy density (Wh kg_{Zn}^{-1})	Current density (mA cm^{-2})	Reference
CoS_2 SSs	640	780		
N-CoS_2 YSSs	744	922	5	This work
Pt/C||RuO_2	788	961		
CoO/N-CNT + NiFe LDH	570	700	20	[50]
NCNT/CoO-NiO/NiCo	594	713	7	[51]
NPMC-1000	735	835	5	[52]
$NiCo_2S_4$/N-CNTs	431.1	554.6	10	[26]
$CoO_{0.87}S_{0.13}$/GN	709	857.9	10	[53]
Ni_3Fe/N-C	528	634	10	[54]
$NiCo_2S_4$/N-CNT	431	555	10	[26]
CoZn-NC-700	578	694	10	[55]
Pt/C + RuO_2	692	892	5	[56]
MnO/Co/PGC	873	1056	5	[57]

Supplementary References

[1] L. Shen, L. Yu, X. Y. Yu, X. Zhang, X. W. Lou, *Angew. Chem. Int. Ed.* **2015**, *54*, 1868.

[2] L. Shen, L. Yu, H. B. Wu, X. Y. Yu, X. Zhang, X. W. Lou, *Nat. Commun.* **2015**, *6*, 6694.

[3] A. Sivanantham, P. Ganesan, S. Shanmugam, *Adv. Funct. Mater.* **2016**, *26*, 4661.

[4] C. Guan, X. Liu, A. M. Elshahawy, H. Zhang, H. Wu, S. J. Pennycook, J. Wang, *Nanoscale Horiz.* **2017**, *2*, 342.

[5] J. Yin, Y. Li, F. Lv, M. Lu, K. Sun, W. Wang, L. Wang, F. Cheng, Y. Li, P. Xi, S. Guo, *Adv. Mater.* **2017**, *29*, 1704681.

[6] J. Hao, W. Yang, Z. Peng, C. Zhang, Z. Huang, W. Shi, *ACS Catal.* **2017**, *7*, 4214.

[7] P. Cai, J. Huang, J. Chen, Z. Wen, *Angew. Chem. Int. Ed.* **2017**, *56*, 4858.

[8] L. Fang, W. Li, Y. Guan, Y. Feng, H. Zhang, S. Wang, Y. Wang, *Adv. Funct. Mater.* **2017**, *27*, 1701008.

[9] X. Han, X. Wu, Y. Deng, J. Liu, J. Lu, C. Zhong, W. Hu, *Adv. Energy Mater.* **2018**, *8*, 1800935.

[10] Y. Hua, H. Jiang, H. Jiang, H. Zhang, C. Li, *Electrochim. Acta* **2018**, *278*, 219.

[11] X. Ma, W. Zhang, Y. Deng, C. Zhong, W. Hu, X. Han, *Nanoscale* **2018**, *10*, 4816.

[12] N. Yao, P. Li, Z. Zhou, R. Meng, G. Cheng, W. Luo, *Small* **2019**, *15*, 1901993.

[13] X. Han, W. Zhang, X. Ma, C. Zhong, N. Zhao, W. Hu, Y. Deng, *Adv. Mater.* **2019**, *31*, 1808281.

[14] W. Xin, W. J. Jiang, Y. Lian, H. Li, S. Hong, S. Xu, H. Yan, J. S. Hu, *Chem. Commun.* **2019**, *55*, 3781.

[15] F. Li, R. Xu, Y. Li, F. Liang, D. Zhang, W.-F. Fu, X.-J. Lv, *Carbon* **2019**, *145*, 521.

[16] R. Xu, Z. Xu, X. Zhang, Y. Ling, M. Li, Z. Yang, *ChemElectroChem* **2020**, *7*, 148.

[17] J.-T. Ren, Z.-Y. Yuan, *ACS Sustainable Chem. Eng.* **2019**, *7*, 10121.

[18] H. Zhao, Y. Yang, X. Dai, H. Qiao, J. Yong, X. Luan, L. Yu, C. Luan, Y. Wang, X. Zhang, *Electrochim. Acta* **2019**, *295*, 1085.

[19] S. Liu, C. Che, H. Jing, J. Zhao, X. Mu, S. Zhang, C. Chen, S. Mu, *Nanoscale* **2020**, *12*, 3129.

[20] M. Chauhan, S. Deka, *ACS Appl. Energy Mater.* **2019**, *3*, 977.

[21] H. Wang, Y. Liang, Y. Li, H. Dai, *Angew. Chem. Int. Ed.* **2011**, *50*, 10969.

[22] C. Zhao, D. Li, Y. Feng, *J. Mater. Chem. A* **2013**, *1*, 5741.

[23] P. Ganesan, M. Prabu, J. Sanetuntikul, S. Shanmugam, *ACS Catal.* **2015**, *5*, 3625.

[24] J. Jiang, S. Lu, H. Gao, X. Zhang, H.-Q. Yu, *Nano Energy* **2016**, *27*, 526.

[25] H. Hu, L. Han, M. Z. Yu, Z. Wang, X. W. Lou, *Energy Environ. Sci.* **2016**, *9*, 107.

[26] X. Han, X. Wu, C. Zhong, Y. Deng, N. Zhao, W. Hu, *Nano Energy* **2017**, *31*, 541.

[27] X. Qiao, J. Jin, H. Fan, Y. Li, S. Liao, *J. Mater. Chem. A* **2017**, *5*, 12354.

[28] W. Liu, J. Zhang, Z. Bai, G. Jiang, M. Li, K. Feng, L. Yang, Y. Ding, T. Yu, Z. Chen, A. Yu, *Adv. Funct. Mater.* **2018**, *28*, 1706675.

[29] L. Guo, J. Deng, G. Wang, Y. Hao, K. Bi, X. Wang, Y. Yang, *Adv. Funct. Mater.* **2018**, *28*, 1804540.

[30] B. Chen, Z. Jiang, L. Zhou, B. Deng, Z.-J. Jiang, J. Huang, M. Liu, *J. Power Sources* **2018**, *389*, 178.

[31] C. Han, Q. Li, D. Wang, Q. Lu, Z. Xing, X. Yang, *Small* **2018**, *14*, 1703642.

[32] W. Liu, B. Ren, W. Zhang, M. Zhang, G. Li, M. Xiao, J. Zhu, A. Yu, L. Ricardez-Sandoval, Z. Chen, *Small* **2019**, *15*, 1903610.

[33] Y. Cao, X. Zheng, H. Zhang, J. Zhang, X. Han, C. Zhong, W. Hu, Y. Deng, *J. Power Sources* **2019**, *437*, 226893.

[34] S. Zhang, D. Zhai, T. Sun, A. Han, Y. Zhai, W.-C. Cheong, Y. Liu, C. Su, D. Wang, Y. Li, *Appl. Catal., B* **2019**, *254*, 186.

[35] Z. Jinyu, S. Liping, K. Fanhao, H. Lihua, Z. Hui, *Int. J. Hydrogen Energy* **2019**, *44*, 3681.

[36] A. Pendashteh, J. S. Sanchez, J. Palma, M. Anderson, R. Marcilla, *Energy Stor. Mater.* **2019**, *20*, 216.

[37] Y. Liang, Q. Gong, X. Sun, N. Xu, P. Gong, J. Qiao, *Electrochim. Acta* **2020**, *342*, 136108.

[38] W. Fang, H. Hu, T. Jiang, G. Li, M. Wu, *Carbon* **2019**, *146*, 476.

[39] L. Li, L. Song, H. Guo, W. Xia, C. Jiang, B. Gao, C. Wu, T. Wang, J. He, *Nanoscale* **2019**, *11*, 901.

[40] S. Dou, L. Tao, J. Huo, S. Wang, L. Dai, *Energy Environ. Sci.* **2016**, *9*, 1320.

[41] Y. Li, J. Yin, L. An, M. Lu, K. Sun, Y.-Q. Zhao, F. Cheng, P. Xi, *Nanoscale* **2018**, *10*, 6581.

[42] Y. Guo, P. Yuan, J. Zhang, Y. Hu, I. S. Amiinu, X. Wang, J. Zhou, H. Xia, Z. Song, Q. Xu, *ACS nano* **2018**, *12*, 1894.

[43] H. Li, Q. Li, P. Wen, T. B. Williams, S. Adhikari, C. Dun, C. Lu, D. Itanze, L. Jiang, D. L. Carroll, *Adv. Mater.* **2018**, *30*, 1705796.

[44] S. Liu, I. S. Amiinu, X. Liu, J. Zhang, M. Bao, T. Meng, S. Mu, *Chem. Eng. J.* **2018**, *342*, 163.

[45] L. Yang, X. Zeng, D. Wang, D. Cao, *Energy Stor. Mater.* **2018**, *12*, 277.

[46] T. Li, Y. Lu, S. Zhao, Z.-D. Gao, Y.-Y. Song, *J. Mater. Chem. A* **2018**, *6*, 3730.

[47] Y. Jiang, Y. P. Deng, J. Fu, D. U. Lee, R. Liang, Z. P. Cano, Y. Liu, Z. Bai, S. Hwang, L. Yang, *Adv. Energy Mater.* **2018**, *8*, 1702900.

[48] B. Q. Li, C. X. Zhao, S. Chen, J. N. Liu, X. Chen, L. Song, Q. Zhang, *Adv. Mater.* **2019**, *31*, 1900592.

[49] L. Li, J. Yang, H. Yang, L. Zhang, J. Shao, W. Huang, B. Liu, X. Dong, *ACS Appl. Energy Mater.* **2018**, *1*, 963.

[50] Y. Li, M. Gong, Y. Liang, J. Feng, J.-E. Kim, H. Wang, G. Hong, B. Zhang, H. Dai, *Nat. Commun.* **2013**, *4*, 1.

[51] X. Liu, M. Park, M. G. Kim, S. Gupta, G. Wu, J. Cho, *Angew. Chem. Int. Ed.* **2015**, *54*, 9654.

[52] J. Zhang, Z. Zhao, Z. Xia, L. Dai, *Nat. Nanotechnol.* **2015**, *10*, 444.

[53] J. Fu, F. M. Hassan, C. Zhong, J. Lu, H. Liu, A. Yu, Z. Chen, *Adv. Mater.* **2017**, *29*, 1702526.

[54] G. Fu, Z. Cui, Y. Chen, Y. Li, Y. Tang, J. B. Goodenough, *Adv. Energy Mater.* **2017**, *7*, 1601172.

[55] B. Chen, X. He, F. Yin, H. Wang, D. J. Liu, R. Shi, J. Chen, H. Yin, *Adv. Funct. Mater.* **2017**, *27*, 1700795.

[56] G. Fu, X. Jiang, Y. Chen, L. Xu, D. Sun, J.-M. Lee, Y. Tang, *NPG Asia Mater.* **2018**, *10*, 618.

[57] X. F. Lu, Y. Chen, S. Wang, S. Gao, X. W. Lou, *Adv. Mater.* **2019**, *31*, 1902339.
